# Supplementary material for: Aberrant Splicing of INS Impairs Beta-Cell Differentiation and Proliferation by ER Stress in the Isogenic iPSC Model of Neonatal Diabetes
Source: Int J Mol Sci. 2022 Aug 8;23(15):8824. doi: 10.3390/ijms23158824 (PMC9369396; doi:10.3390/ijms23158824)
Supplement: Supplementary file 1 [file ijms-23-08824-s001.zip › ijms-1830102-supplementary.pdf]

International Journal of
Molecular Sciences

MDPI

Article

Aberrant splicing of *INS* impairs beta-cell differentiation and proliferation by ER stress in the isogenic iPSC model of neonatal diabetes

Alexandra V. Panova, Natalia V. Klementieva, Anna V. Sycheva, Elena V. Korobko, Anastasia O. Sosnovtseva, Tatiana S. Krasnova, Maria R. Karpova, Petr M. Rubtsov, Yulia V. Tikhonovich, Anatoly N. Tiulpakov, Sergey L. Kiselev

Table S1. List of sgRNAs and oligonucleotides for sgRNA cloning.

Name	Sequence, 5′-3′	PAM	Oligonucleotide sequence for cloning
sgRNA-1	GTTCCAGAACCTGCTCTGCG	CGG	Fwd: GTTCCAGAACCTGCTCTGCGGTTTT<br>Rev: CGCAGAGCAGGTTCTGGAACCGGTG
sgRNA-2	CAGGTTCTGGAACAGCGGCG	AGG	Fwd: CAGGTTCTGGAACAGCGGCGGTTTT<br>Rev: CGCCGCTGTTCCAGAACCTGCGGTG
sgRNA-3	CAGAGCAGGTTCTGGAACAG	CGG	Fwd: CAGAGCAGGTTCTGGAACAGGTTTT<br>Rev: CTGTTCCAGAACCTGCTCTGCGGTG

Single-stranded oligodeoxynucleotide (ssODN):

5′-TCCTGTGTCCCTCTGCCTCGCCGCTGTTCCGGAACCTGCTCTGCGCGGCACGTCCTGGCAG-TCGGGCAGGTGGAGCTGGGCGGGGGCCCTGGT-3′

Table S2. Comparison of culture conditions for iPSC single cell cloning after FACS analysis.

Matrix	Medium	Viability, %
Feeder cells on Matrigel	mTESR1	2.1%
Feeder cells on Matrigel	½ mTESR1 + ½ conditioned mTESR1	5.2%
Feeder cells on Matrigel	½ mTESR1 + ½ conditioned mTESR1 + 10% KSR	8.3%
Matrigel	½ mTESR1 + ½ conditioned mTESR1	0%

Table S3. Summary of sequencing results of iPSC clone screening.

Clone number	Intron genotype	Presence of cleavage
patient	Heterozygous mutation c.188-31G>A	N/A
	**sgRNA-3**	

3	Heterozygous mutation c.188-31G>A	no
13	Heterozygous mutation c.188-31G>A	no
16	Heterozygous mutation c.188-31G>A	no
17	Heterozygous mutation c.188-31G>A	no
18	Del/ins at the position c.188-31G>A	yes
27	Heterozygous mutation c.188-31G>A	no
42	Heterozygous mutation c.188-31G>A	no
2	Heterozygous mutation c.188-31G>A	no
3	Del/ins at the position c.188-31G>A	yes
4	Heterozygous mutation c.188-31G>A	no
6	Heterozygous mutation c.188-31G>A	no
7	Del/ins at the position c.188-31G>A	yes
9	Del/ins at the position c.188-31G>A	yes
10	Heterozygous mutation c.188-31G>A	no
11	Heterozygous mutation c.188-31G>A	no
12	Heterozygous mutation c.188-31G>A	no
13	Heterozygous mutation c.188-31G>A	no
14	Heterozygous mutation c.188-31G>A	no
15	Heterozygous mutation c.188-31G>A	no
16	Heterozygous mutation c.188-31G>A	no
18	Del/ins at the position c.188-31G>A	yes
19	Heterozygous mutation c.188-31G>A	no
20	Heterozygous mutation c.188-31G>A	no
21	Heterozygous mutation c.188-31G>A	no
22	Del/ins at the position c.188-31G>A	yes
	**sgRNA-2**	
1	Heterozygous mutation c.188-31G>A	no
2	Heterozygous mutation c.188-31G>A	no

3	Del/ins at the position c.188-31G>A	yes
4	Heterozygous mutation c.188-31G>A	no
5	Heterozygous mutation c.188-31G>A	no
6	Heterozygous mutation c.188-31G>A	no
7	Heterozygous mutation c.188-31G>A	no
8	Heterozygous mutation c.188-31G>A	no
9	Heterozygous mutation c.188-31G>A	no
10	Heterozygous mutation c.188-31G>A	no
11	Heterozygous mutation c.188-31G>A	no
12	Del/ins at the position c.188-31G>A	yes
13	Heterozygous mutation c.188-31G>A	no
14	Heterozygous mutation c.188-31G>A	no
15	Heterozygous mutation c.188-31G>A	no
16	Heterozygous mutation c.188-31G>A	no
17	Del/ins at the position c.188-31G>A	yes
18	Heterozygous mutation c.188-31G>A	no
19	Duplication	yes
20	Insertion 7bp	yes
21	Heterozygous mutation c.188-31G>A	no
22	Heterozygous mutation c.188-31G>A	no
23	Heterozygous mutation c.188-31G>A	no
24	Heterozygous mutation c.188-31G>A	no
36	Heterozygous mutation c.188-31G>A	no
37	Del/ins at the position c.188-31G>A	yes
38	Heterozygous mutation c.188-31G>A	no
39	Heterozygous mutation c.188-31G>A	no
40	Heterozygous mutation c.188-31G>A	no
41	Heterozygous mutation c.188-31G>A	no

46	Del/ins at the position c.188-31G>A	yes
47	Heterozygous mutation c.188-31G>A	no
48	Heterozygous mutation c.188-31G>A	no
49	Heterozygous mutation c.188-31G>A	no
50	Del/ins at the position c.188-31G>A	yes
51	Heterozygous mutation c.188-31G>A	no
52	Del/ins at the position c.188-31G>A	yes
53	Heterozygous mutation c.188-31G>A	no
54	Heterozygous mutation c.188-31G>A	no
55	Del/ins at the position c.188-31G>A	yes
56	Heterozygous mutation c.188-31G>A	no
57	Heterozygous mutation c.188-31G>A	no
58	Heterozygous mutation c.188-31G>A	no
59	Heterozygous mutation c.188-31G>A	no
60	Heterozygous mutation c.188-31G>A	no
61	Homozygous c.188-31G	yes
62	Heterozygous mutation c.188-31G>A	no
63	Heterozygous mutation c.188-31G>A	no
64	Heterozygous mutation c.188-31G>A	no
65	Heterozygous mutation c.188-31G>A	no

Table S4. Key points of pancreatic differentiation protocol.

**Stage 1**	Day 1	Basa medium + supplement A + supplement B
	Day 2	Basal medium + supplement B
	Day 3	
	Day 4	

**Stage 2 (posterior foregut induction)**	Day 5<br>Day 6	RPMI + B27 (1×)<br><br>+FGF7 50ng/ml
**Stage 3 (pancreatic endoderm induction)**	Day 7<br>Day 8<br>Day 9	DMEM, Glutamax (1x), sodium pyruvate (1x), B27 (1×)<br><br>+KAAD 0.25μM<br>+RA 2μM<br>+LDN 250nM
**Stage 4 (pancreatic progenitor induction)**	Day 10<br>Day 11<br>Day 12	DMEM, Glutamax (1x), sodium pyruvate (1x), B27 (1×)<br><br>+EGF 50ng/ml
**Stage 5**	Day 13<br>Day 14	DMEM, Glutamax (1x), sodium pyruvate (1x), B27 (1×)<br><br>+ KAAD 0.25μM<br>+ T3 1μM<br>+ Alk5i 10μM<br>+ Zinc-Sulfate 10μM<br>+ Heparin 10μg/ml<br>+ Y-27632 5μM

Table S5. List of reagents for beta-cell culturing.

Reagent	Company	Final concentration	Stock concentration
Sodium bicarbonate	Merck	1.5 g/l	150g/L (x100)
BSA	Gibco	2%	100 %
SANT-1	Millipore	0.25 μM	0,25mM (x1000)

Retinoic acid	Stemcell Technologies	0.05 µM	1mM (x20.000)<br>Or 50 mkM (x1000)
LDN193189	Stemcell Technologies	100 nM	1mM (x10.000)
ITS-X	Gibco	1:200	100% (x200)
T3	Millipore	1 µM	1mM (x1000)
ALK5 inhibitor II	Abcam	10 µM	10 mM (x1000)
Heparin	Sigma-Aldrich	10 µg/ml	10 mg/ml (x1000)
EGF	R&D Systems	50 ng/ml	500 µg/ml (x10.000)
Cyclopamine	Stemcell Technologies	0,25 µM	5mM (x20.000)
KGF (FGF7)	Abcam	50 ng/ml	100 µg/ml (x2.000)
B27	Gibco	1:100	100% (x100)

Table S6. List of primers for the *INS* splice variant isoform cloning.

Name	Sequence, 5′-3′
Ins-CDS-RI-F	ATCGAATTCACTGTCCTTCTGCCATGGCCCTG
R1InsEx3-R	TTGAATTCCATCTCTCTCGGTGCAGG
GRP78-LucF	TCCGGTACCCGAGATAGACAGCTGCTGAACCA
Grp78-LucR	TTCCCTCGAGCTTCATCTTGCCAGCCAGT
UPRE-1-For	CACAGGTGCTGACGTGGCATTCACAGGTGCTGACGTGGCATTCACAGGT
UPRE-1-Rev	GTGAATGCCACGTCAGCACCTGTGAATGCCACGTCAGCACCTGTGGTAC
UPRE-2-For	GCTGACGTGGCATTCACAGGTGCTGACGTGGCATTCACAGGTGCTGACGTGGCATTC
UPRE-2-Rev	TCGAGAATGCCACGTCAGCACCTGTGAATGCCACGTCAGCACCTGTGAATGCCACGTCAGCACCT
ERSE-For	CTTCACCAATCGGCGGCCTCCACGACGGC
ERSE-Rev	TCGAGCCGTCGTGGAGGCCGCCGATTGGTGAAGGTAC

Table S7. Primers for iPSC clone screening after CRISPR/Cas9 genome editing (371-bp PCR product).

	Sequence, 5′-3′	Tm	GC %
Forward	GGGGAGAAGTACTGGGATCACC	61.55	59.09
Reverse	ATTGTTCCACAATGCCACGCTT	61.92	45.45

Table S8. List of antibodies for immunocytochemistry.

Antibody	Company, catalog number	Dilution
Ms anti-PDX1 PE-conjugated	BD Biosciences, 562161	1:100
Ms anti-NKX6.1 Alexa Fluor 647-conjugated	BD Biosciences, 563338	1:100
Rb mAb to Insulin, Alexa Fluor 647-conjugated	Cell Signaling Technology, 9008	1:500
Rb pAb to C-Peptide	Cell Signaling Technology, 4593	1:100
Ms pAb to Glucagon	Sigma, 62654	1:1000
Ms mAb to Somatostatin	Santa Cruz SC-55565	1:50
Goat anti-Mouse IgG H&L, Alexa Fluor 488	Abcam, ab150117	1:500
Goat anti-Mouse IgG H&L, Alexa Fluor 594	Abcam, ab150120	1:500
Goat anti-Rabbit IgG H&L, Alexa Fluor 488	Abcam, ab150077	1:500
Goat anti-Rabbit IgG H&L, Alexa Fluor 594	Abcam, ab150084	1:500

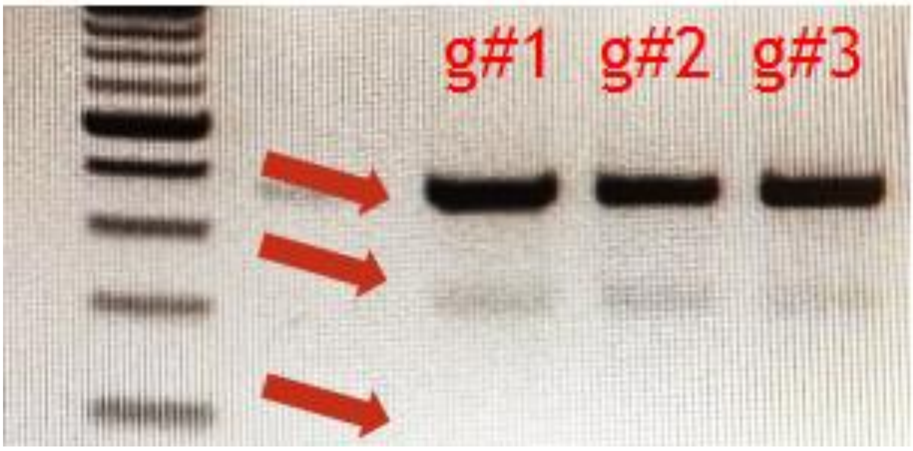

Figure S1. Analysis of cutting efficiency of the *INS* allele-specific sgRNAs by T7E1 assay. sgRNA-1, sgRNA-2 and sgRNA-3 used in our study are marked as g#1, g#2 and g#3 on the electrophoregram. Red arrows show bands which are generated when Cas9 nuclease makes modifications to genomic DNA. The cutting efficiency was calculated based on band intensity and amounted to 10%, 11.1%, and 8.9% for sgRNA-1, sgRNA-2 and sgRNA-3, respectively.

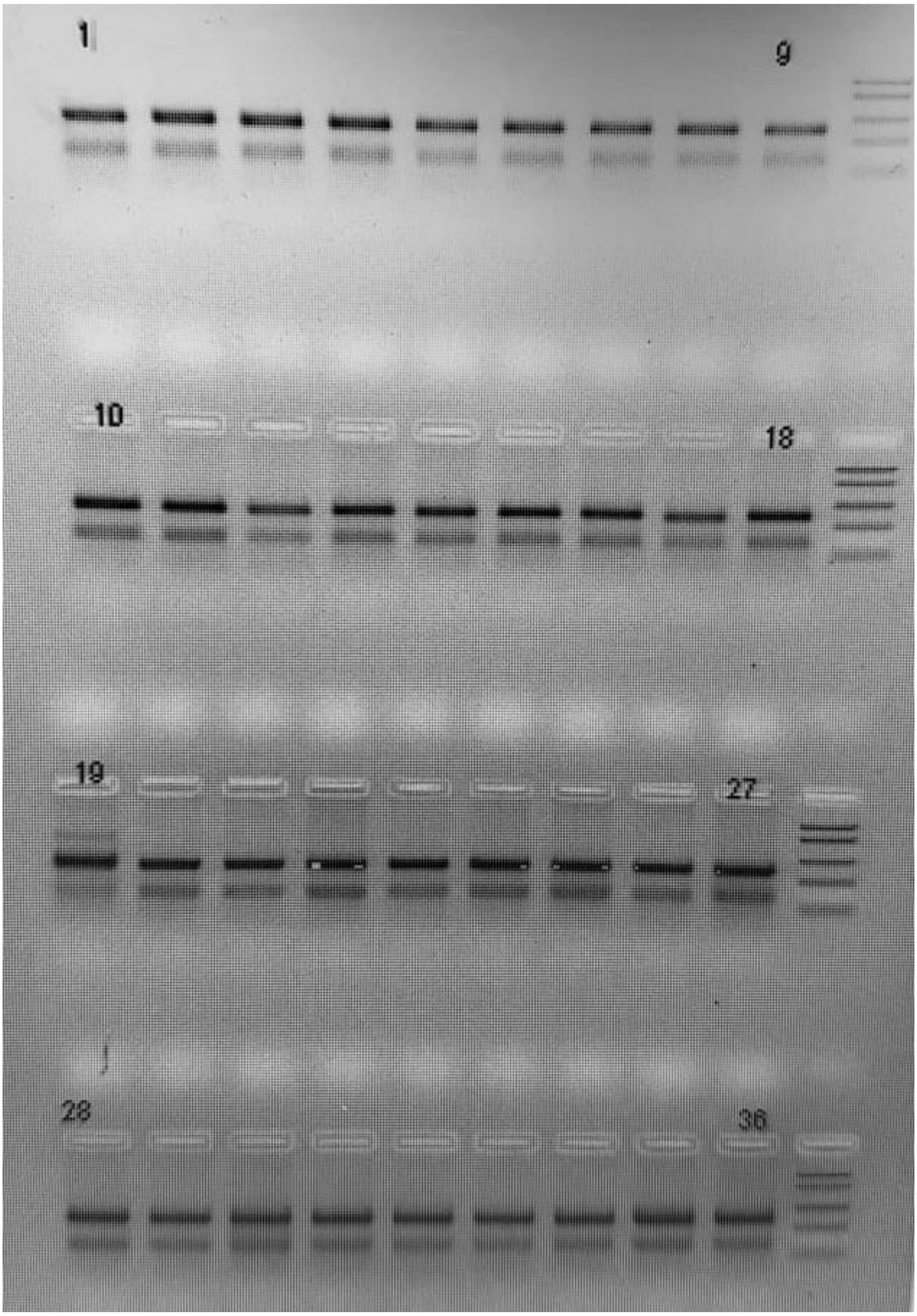

Figure S2. Restriction digest screening of iPSC clones after CRISPR/Cas9 editing using *BtsI* enzyme. The electrophoregram shows screening results for the first 36 clones, wherein an additional 400-bp band expected in the case of HDR is observed in clone #19.

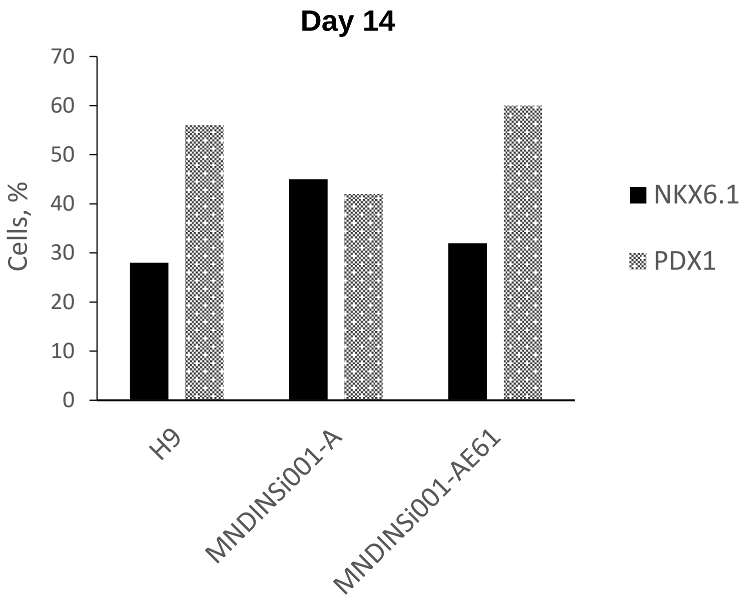

Figure S3. The capacity of established isogenic iPSC lines to differentiate into pancreatic progenitor cells. The data show the amount of NKX6.1- and PDX1-positive cells at Day 14 of differentiation for MNDINSi001-A iPSC line carrying c.188-31G>A *INS* mutation and CRISPR/Cas9-corrected MNDINSi001-AE61 iPSC line. H9 ESC line was used as a gold-standard.
